# Metabolic changes in an animal model of amyotrophic lateral sclerosis evaluated by [^18^F]-FDG positron emission tomography

**DOI:** 10.1186/s40035-021-00246-1

**Published:** 2021-06-23

**Authors:** Bruno Lima Giacobbo, Tomás Mediavilla, Daniel J. Marcellino, Fahad Sultan

**Affiliations:** Department of Integrative Medical Biology, Umeå University, Umeå, Sweden

## Main text

Energy metabolism has been proposed to be affected in amyotrophic lateral sclerosis (ALS), due to its relationship with SOD1^G93A^-dependent mitochondrial loss-of-function [1, 2]. Although the metabolic state and the use of its dysfunction as an early diagnostic criterion have been evaluated in ALS patients by positron emission tomography (PET), preclinical research in animal models is rare. In this study, we imaged glucose metabolism using [^18^F]-fluorodeoxyglucose (FDG) (NanoScan PET/CT, Mediso Medical Imaging Systems, Hungary) in both male and female mice expressing a mutated human SOD1 variant (hSOD1^G93A^) with ALS-like symptoms, and compared these animals to age- and sex-matched littermate controls (SOD1^WT^).

**Fig. 1:**
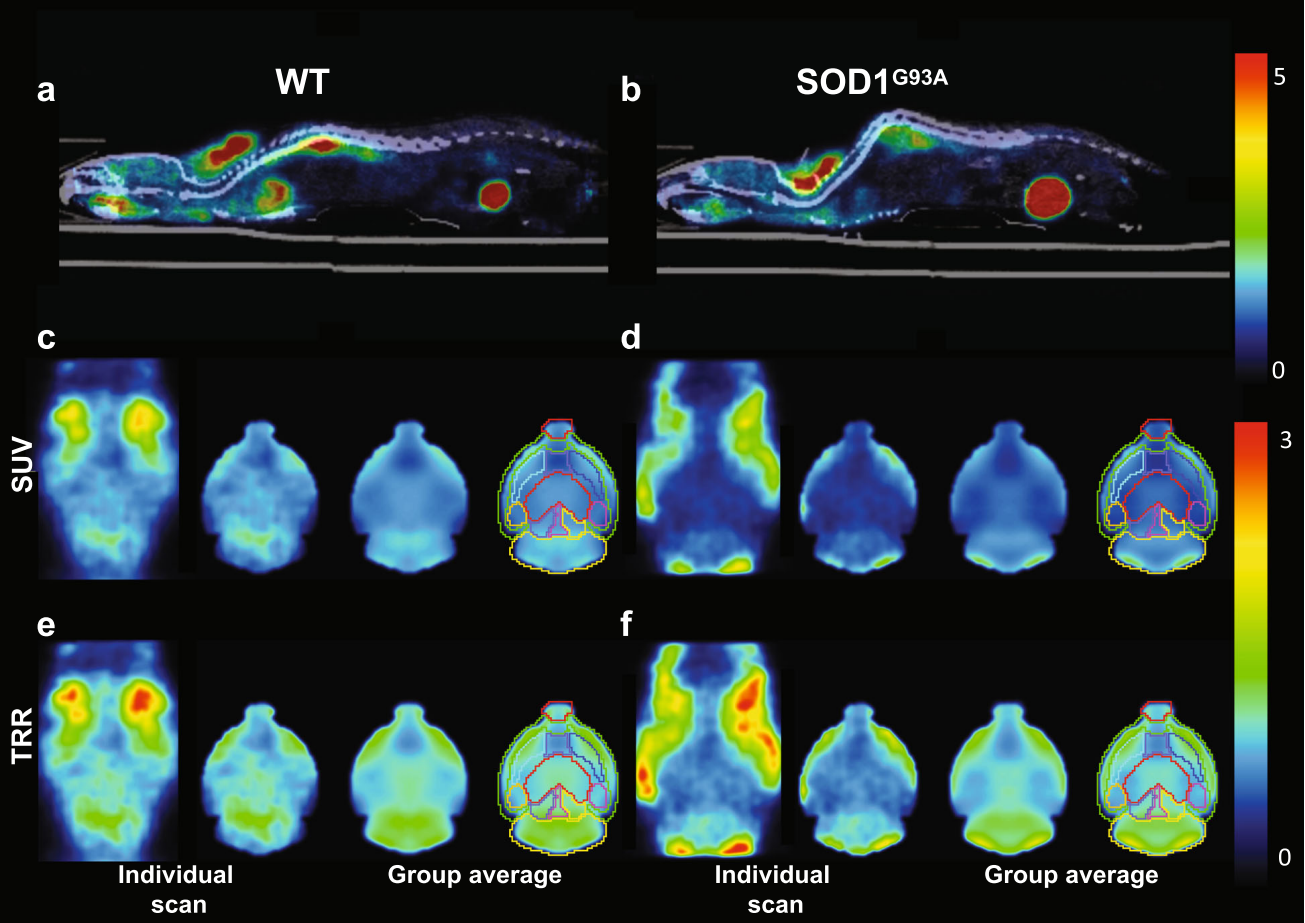
**a, b:** Representative images of whole-body [^18^F]-FDG at the sagittal plane in a WT (**a**) and an ALS animal (**b**). Color scale bar indicates radiotracer uptake value normalized for injected dose and weight (SUV). **c**, **d**: Horizontal plane of the head in cropped SUV images. From left to right: an individual head-cropped image; an individual brain-masked image; group-based average image; and volume of influence (VOI) placement for data analysis. Color scale bar indicates radiotracer uptake normalized for injected dose and weight (SUV). **e**, **f**: Horizontal plane of the head with radioactivity normalized to the average radioactivity in the whole brain (TRR). From left to right: an individual head-cropped image; an individual brain-masked image; group-based average image; and VOI placement for data analysis. Color scale bar indicates radiotracer uptake normalized for averaged radioactivity in the whole brain

Generalized Linear Models analysis (Supplemental Methods) showed significant between-group differences in body weight at the time of scan (*F* = 28.81; *P* < 0.0001), with post-hoc comparisons indicating that the male WT mice weighed significantly higher than all other groups, while the female ALS mice weighed significantly lower when compared with all other groups (Table S1). There were no significant differences in the age or the injected dose administered at the time of scan (all *P* > 0.05). The [^18^F]-FDG uptake showed a marked difference between WT and ALS mice (Fig. 1). There was a lower uptake of [^18^F]-FDG in the brains of ALS mice compared to the WT littermates. A detailed statistical analysis of standardized uptake value (SUV)-normalized [^18^F]-FDG uptake using a Generalized Estimating Equations approach revealed a significant effect of sex for almost all regions evaluated (Table S2), with female mice showing a higher SUV for all regions assessed (main effect of sex: Wald-*χ*^2^ < 0.0001) except the cerebellum, basal forebrain and amygdala that showed no significant sex difference. We observed a significant effect of genotype on the [^18^F]-FDG SUV uptake (main effect of genotype: Wald-*χ*^2^ < 0.0001) with significantly lower SUV uptake in the hippocampus, thalamus, midbrain, and right inferior colliculus in SOD1^G93A^ than in the WT animals. In general, there was a significant interaction effect between sex and genotype (sex × genotype interaction: Wald-*χ*^2^ < 0.0001). Pairwise comparisons revealed significant differences between WT females and SOD1^G93A^ males in the left hippocampus (*P* < 0.0001) and the left midbrain (*P* = 0.049).

Due to a possible influence of body weight between ALS and WT mice, [^18^F]-FDG uptake from each volume of influence (VOI) was normalized to the average uptake of the whole brain for each individual, to eliminate any possible effect of body weight. The tissue-to-reference ratio (TRR) analysis showed that the sex differences found in SUV disappeared except in the right striatum. Regarding the main effect of genotype, there were significant differences between the WT and SOD1^G93A^ animals in the hippocampus, thalamus, basal forebrain, olfactory bulb, right amygdala, midbrain, and right inferior colliculus (Table S3).

In this study, we found significant changes in glucose metabolism in the brains of SOD1^G93A^ animals when compared with WT control littermates, indicating a change in metabolic pattern in these animals. To specifically investigate early changes in the central nervous system, we scanned animals that were at an initial stage of disease, stage 1 [3].

The effects of ALS on metabolic function in humans and animal models of ALS are yet to be fully understood. In animals, one study has measured the glucose uptake in the brains of mice carrying the human TDP-43^A315T^ mutation [4]. The authors observed a significant hypometabolism in motor and somatosensory cortex, as well as a hypermetabolism in amygdala and brainstem. In humans, however, brain metabolism in ALS patients with [^18^F]-FDG PET has been studied with a focus on prediction of ALS onset and characterization of disease progression, both with mixed results [5]. In humans, PET [^18^F]-FDG studies have reported a widespread change in brain glucose uptake. Some of these studies have pointed towards a decrease in [^18^F]- FDG PET uptake in primary motor and other premotor regions, while also showing hypermetabolic regions such as the limbic system, brainstem and cerebellum, irrespective of the ALS onset type (i.e. bulbar- or spinal-onset ALS) [6]. The hypometabolism in frontal regions has been explained with the “dying forward” hypothesis of ALS [7] leading to a reduction in glutamatergic cortico-cortical neurotransmission, which has a major effect on glucose consumption. Our observation of early reductions in glucose uptake suggests that hypometabolism can also occur prior to such changes in neurotransmission and can be due to an overall hypometabolic state in ALS.

Hypometabolism has also been described in other brain regions in humans, such as in the thalamus [5]. Consistently, we found a 12% decrease in thalamic [^18^F]-FDG PET uptake normalized to the whole brain. Interestingly, these changes in thalamic glucose uptake appear to be specific for familial ALS as recently proposed in a study of carriers of C9orf72 gene [8]. However, it is not entirely clear whether specific thalamic nuclei play a key role in the onset of ALS, or whether the hypometabolism of thalamus is associated with overall reductions in the relay of motor and sensory information throughout the brain.

Further, we observed bilateral decreases of metabolic activity in hippocampus and midbrain in SOD1^G93A^ mice, in both SUV and TRR (Tables S2 and S3). Interestingly, human data predominantly report a hypermetabolic state in hippocampus and midbrain, either by PET or through the use of additional in vivo techniques [8]. An explanation for such increased metabolism in these areas is an increase of reactive gliosis from oxidative stress and neurotoxicity. Structural MRI data, on the other hand, suggest a relative decrease in hippocampal volume in ALS patients and loss of hippocampus-dependent cognitive functions [9], with similar atrophy observed in midbrain. This atrophy in the midbrain and hippocampus has also been observed in SOD1^G93A^ animals. Thus, it is possible that this increased atrophy in the midbrain is also related to a decreased glucose metabolism resulting from neuronal loss of function and neurodegeneration. In fact, a study has reported increased gliosis in hippocampus and brainstem of SOD1^G93A^ rats prior to the development of ALS-like symptomatology [10], suggesting that the hypermetabolic state of hippocampus might occur at earlier stages of disease, and then decline over time to a hypometabolic state as disease progresses. In the future, longitudinal scans including those at a prodromal stage and at several time points later in ALS animal models are needed to test this hypothesis.

## Supplementary Information

**Additional file 1: Supplementary methods.** Detailed description of material and methods.

**Additional file 2: Supplementary tables: Table S1.** Animal weight, radioactivity measurements and animal age at the time of scan. **Table S2.** Main effects and interaction of sex and genotype in [F]-FDG SUV uptake. **Table S3.** Main effects and interaction of sex and genotype in [^18^F]-FDG activity normalized to the Whole Brain (TRR).

## Data Availability

The datasets used during the current study are available from the corresponding author on reasonable request.

## Abbreviations

ALS: Amyotrophic lateral sclerosis
[^18^F]-FDG: [^18^F]-fluorodeoxyglucose
PET: Positron emission tomography
SUV: Standardized uptake values
TRR: Tissue to reference ratio
VOI: Volume of influence
