# Supplementary material for: Metabolic changes in an animal model of amyotrophic lateral sclerosis evaluated by [18F]-FDG positron emission tomography
Source: Transl Neurodegener. 2021 Jun 23;10:21. doi: 10.1186/s40035-021-00246-1 (PMC8220836; doi:10.1186/s40035-021-00246-1)
Supplement: Supplementary file 1 — Additional file 1: Supplementary methods. Detailed description of material and methods. [file 40035_2021_246_MOESM1_ESM.docx]

# Supplementary methods

## Animals

All experimental procedures were conducted in accordance with Umeå University animal ethical committee (Ethical permit number: 5.2.18-19236/17). C57Bl6/J mice expressing human SOD1^G93A^ were kindly donated by Thomas Brännström and originated from the JAX™ B6.Cg-Tg(SOD1*G93A)1Gur/J strain (stock number 004435). Animal breeding was maintained at the experimental animal facility at the Umeå Centre for Comparative Biology (UCCB) under SPF conditions. hSOD1^G93A^ genotyping was performed at the Umeå Transgene Core Facility (UTCF) by PCR of genomic DNA from ear tissue removed after ear marking. After genotyping, animals were divided between ALS (hSOD1^G93A^) and wild-type (SOD1^WT^). Animals were group housed and maintained under controlled temperature, humidity, and 12-h day/night cycle.

The criteria of ALS symptom progression used in this study is similar to those developed by Hatzipetros and colleagues (Hatzipetros et al 2015). Briefly, the ALS phenotype is divided as follows: stage 0) where animals show no apparent ALS-like symptoms; stage 1) where animal locomotion is slightly impaired, but functions are normal. When an animal is suspended by the tail it does not sustain an outward splay of the hind limbs; stage 2) where animal locomotion is impaired, showing paresis of hind limbs; and stage 3) when animal is placed on its side, it is unable to right itself or takes longer than 5 seconds to do so. In ALS hSOD1^G93A^ mice stage 1 occurs 4 weeks before the beginning of stage 3 (Hatzipetros et al 2015). Stage 3 was considered the humane endpoint and animals reaching this stage were terminated.

## [^18^F]-FDG data acquisition and analysis

When SOD1^G93A^ animals reached a score of 1 for ALS symptoms, as explained above, animals were submitted to a static, 10 minute long [^18^F]-FDG scan to evaluate glucose uptake in the brain. Animals were fasted for four hours prior to injection of [^18^F]-FDG. After the period of fasting, mice were weighed and then placed under deep anesthesia using isoflurane (4% induction, 2% maintenance). For [^18^F]-FDG administration, a cannula was inserted into the tail vein and radiotracer was administered as a bolus injection (11.23 ± 2.01 MBq) with the subsequent flushing of the cannula using sterile saline (Braun, 0.9% NaCl). Isoflurane was withdrawn and animals were allowed to wake and move freely for 60 minutes in their home cage until the time of the scan (mean radioactivity at the time of scan: 7.40 ± 1.57 MBq - see Table 1). Animals were anesthetized and placed on a heated bed and [^18^F]-FDG was imaged (NanoScan PET/CT, Mediso Medical Imaging Systems, Hungary). A 5-minute computerized tomography (CT) was performed prior to PET imaging for scattering and attenuation correction. Static PET images were acquired in one frame of ten minutes, after which the animal was removed from bed and allowed to wake up before being returned to its home cage. Age- and sex-matched SOD1^WT^ littermates were submitted to the identical imaging procedure as SOD1^G93A^ mice and were used as controls.

Data was iteratively reconstructed using the Maximum-Likelihood-Expectation-Maximization (MLEM) provided by the manufacturer software (TeraTomo, Mediso Medical Imaging Systems, Hungary). Nominal resolution at the center of the Field of view is 0.7 mm, and 0.8 mm at 3 cm. The resulting image was 200x200x490 voxels in total with a voxel size of 0.2mm. Each [^18^F]-FDG image of the brain was cropped and the images were aligned using SPM12. An averaged image of all the scans acquired was calculated using SPM12 toolbox SAMIT (Vállez Garcia et al 2015) and the resulting template was coregistered to a mouse atlas using PMOD 4.1 (PMOD Technologies Inc., Switzerland) (Mirrione et al 2007). A volume of influence (VOI)-based correction for partial volume effects (PVC) was performed using Geometric Transfer Matrix (GTM) for the regions of interest to avoid spillover effects from the surrounding head and neck muscles. The average radioactivity values were normalized for weight and injected dose to determine standardized uptake values (SUV). Due to large differences in body weight and fat composition between males and females, and between SOD1 and WT animals, an additional value was obtained by normalizing the radioactivity of each VOI by the average radioactivity in the entire brain (Tissue to reference ratio: TRR).

## Statistical analysis

Data regarding weight, age and injected dose were analyzed using Generalized Linear Models (GLM), with sex (Male x Female), genotype (SOD1^G93A^ x SOD1^WT^) as factors, as well as the interaction between both factors. Both SUV and TRR for each brain region were analyzed using Generalized Estimating Equations (GEE) using a similar multifactorial approach as with GLM but allowing for a more robust comparison between groups at the evaluated regions. All data regarding interactions between sex and genotype were corrected for multiple comparisons using the Bonferroni multiple comparison method. A p-value smaller than 0.05 was used to indicate statistical significance. All data was analyzed using SPSS version 26 (IBM, Newark, U.S.).

## Supplementary references

Hatzipetros T, Kidd JD, Moreno AJ, Thompson K, Gill A, Vieira FG. 2015. A Quick Phenotypic Neurological Scoring System for Evaluating Disease Progression in the SOD1-G93A Mouse Model of ALS. *J Vis Exp*

Mirrione MM, Schiffer WK, Fowler JS, Alexoff DL, Dewey SL, Tsirka SE. 2007. A novel approach for imaging brain-behavior relationships in mice reveals unexpected metabolic patterns during seizures in the absence of tissue plasminogen activator. *Neuroimage* 38: 34-42

Vállez Garcia D, Casteels C, Schwarz AJ, Dierckx RAJO, Koole M, Doorduin J. 2015. A standardized method for the construction of tracer specific PET and SPECT rat brain templates: Validation and implementation of a toolbox. *PLoS ONE* 10: 1-21
