# Supplementary material for: Metabolic changes in an animal model of amyotrophic lateral sclerosis evaluated by [18F]-FDG positron emission tomography
Source: Transl Neurodegener. 2021 Jun 23;10:21. doi: 10.1186/s40035-021-00246-1 (PMC8220836; doi:10.1186/s40035-021-00246-1)
Supplement: Supplementary file 2 — Additional file 2: Supplementary tables: Table S1. Animal weight, radioactivity measurements and animal age at the time of scan. Table S2. Main effects and interaction of sex and genotype in [F]-FDG SUV uptake. Table S3. Main effects and interaction of sex and genotype in [18F]-FDG activity normalized to the Whole Brain (TRR). [file 40035_2021_246_MOESM2_ESM.docx]

**Table S1:** Animal weight, radioactivity measurements and animal age at the time of scan.

|  |  | Weight (g) | Injected dose (MBq) | Age at time of scan  (days) |
| --- | --- | --- | --- | --- |
| SOD1^G93A^ (*n=*8) | Females (*n=*4) | 21.025 ± 1.028* | 6.653 ± 1.94 | 212.5 ± 9.95 |
|  | Males  (*n=*4) | 25.675 ± 1.509 | 8.38 ± 1.605 | 204.5 ± 10.97 |
| SOD1^WT^ (*n=*8) | Females (*n=*4) | 25.55 ± 0.94 | 7.224 ± 1.508 | 212 ± 5.831 |
|  | Males  (*n=*4) | 31.9 ± 2.62* | 7.355 ± 1.594 | 212 ± 22.045 |

* p<0.05 when compared with all other groups. Data are expressed as Mean ± SD.

**Table S2**: Main effects and interaction of sex and genotype in [18F] FDG SUV uptake.

|  | **SOD1^WT^ (*n=*8)** | | **SOD1^G93A^ (*n=*8)** | | **Main effects and Interaction** | | | | |
| --- | --- | --- | --- | --- | --- | --- | --- | --- | --- |
|  | **Females**  ***n=*4** | **Males**  ***n=*4** | **Females**  ***n=*4** | **Males**  ***n=*4** | **% change from Males** | **Sig. Sex** | **% change from WT** | **Sig. Genotype** | **Interaction Sex x Genotype** |
| **Right Striatum** | 1.968 ± 0.042 | 1.489 ± 0.138 | 1.526 ± 0.102 | 1.404 ± 0.095 | 17.23 | **0.001** | -17.96 | 0.27 | 0.645 |
| **Left Striatum** | 1.924 ± 0.047 | 1.523 ± 0.137 | 1.471 ± 0.117 | 1.447 ± 0.091 | 12.53 | **0.005** | -18.15 | 0.151 | 0.449 |
| **Cortex** | 2.179 ± 0.062 | 1.819 ± 0.115 | 1.778 ± 0.075 | 1.721 ± 0.076 | 10.55 | **0.013** | -14.27 | 0.448 | 0.947 |
| **Right Hippocampus** | 1.645 ± 0.026 | 1.3 ± 0.103 | 1.181 ± 0.134 | 1.149 ± 0.064 | 13.36 | **0.004** | -26.43 | **0.014** | 0.41 |
| **Left Hippocampus** | 1.746 ± 0.06 | 1.297 ± 0.112 | 1.172 ± 0.124 | 1.241 ± 0.067 | 13.04 | **0.003** | -26.15 | **0.008** | **0.043** |
| **Thalamus** | 1.663 ± 0.073 | 1.267 ± 0.113 | 1.135 ± 0.114 | 1.178 ± 0.069 | 12.65 | **0.007** | -26.71 | **0.012** | 0.12 |
| **Cerebellum** | 1.841 ± 0.237 | 1.675 ± 0.233 | 1.595 ± 0.028 | 1.887 ± 0.172 | -3.65 | 0.757 | -0.98 | 0.602 | 0.639 |
| **Basal Forebrain** | 1.43 ± 0.065 | 1.158 ± 0.094 | 1.467 ± 0.068 | 1.39 ± 0.177 | 12.04 | 0.089 | 9.41 | 0.138 | 0.823 |
| **Hypothalamus** | 1.003 ± 0.039 | 0.848 ± 0.048 | 0.793 ± 0.055 | 0.746 ± 0.042 | 11.25 | **0.046** | -20.23 | 0.345 | 0.828 |
| **Right Amygdala** | 0.999 ± 0.028 | 0.925 ± 0.065 | 1.212 ± 0.163 | 1.212 ± 0.091 | 3.37 | 0.392 | 20.66 | 0.065 | 0.609 |
| **Left Amygdala** | 0.889 ± 0.034 | 0.846 ± 0.012 | 0.862 ± 0.073 | 0.912 ± 0.062 | -0.4 | 0.461 | 2.23 | 0.352 | 0.704 |
| **Brainstem** | 1.411 ± 0.072 | 1.121 ± 0.03 | 1.164 ± 0.087 | 1.167 ± 0.096 | 11.16 | **0.011** | -8.64 | 0.868 | 0.502 |
| **Central Gray** | 1.805 ± 0.038 | 1.289 ± 0.15 | 1.243 ± 0.142 | 1.335 ± 0.135 | 13.91 | **0.018** | -20.01 | 0.162 | 0.077 |
| **Superior Colliculus** | 1.65 ± 0.025 | 1.297 ± 0.118 | 1.202 ± 0.13 | 1.267 ± 0.067 | 10.12 | **0.012** | -19.38 | 0.064 | 0.13 |
| **Olfactory Bulb** | 2.633 ± 0.062 | 2.17 ± 0.151 | 1.996 ± 0.107 | 1.985 ± 0.127 | 10.24 | **0.008** | -20.68 | 0.069 | 0.588 |
| **Right Midbrain** | 1.643 ± 0.115 | 1.208 ± 0.107 | 1.066 ± 0.116 | 1.013 ± 0.119 | 18 | **0.004** | -37.12 | **0.003** | 0.255 |
| **Left Midbrain** | 1.792 ± 0.123 | 1.246 ± 0.107 | 1.137 ± 0.144 | 1.2 ± 0.064 | 16.51 | **0.002** | -30 | **0.004** | **0.016** |
| **Left Inferior Colliculus** | 2.336 ± 0.114 | 1.672 ± 0.21 | 1.618 ± 0.221 | 1.694 ± 0.216 | 14.87 | **0.039** | -21.02 | 0.218 | 0.179 |
| **Right Inferior Colliculus** | 2.337 ± 0.064 | 1.839 ± 0.18 | 1.452 ± 0.19 | 1.563 ± 0.097 | 10.22 | **0.039** | -38.49 | **0.001** | 0.133 |

Data are expressed as mean ± SEM.

**Table S3**: Main effects and interaction of sex and genotype in [18F] FDG activity normalized to the Whole Brain (TRR).

|  | **SOD1^WT^ (*n=*8)** | | **SOD1^G93A^ (*n=*8)** | | **Main effects and Interaction** | | | | |
| --- | --- | --- | --- | --- | --- | --- | --- | --- | --- |
|  | **Females**  ***n=*4** | **Males**  ***n=*4** | **Females**  ***n=*4** | **Males**  ***n=*4** | **% change from Males** | **Sig. Sex** | **% change from WT** | **Sig. Genotype** | **Interaction Sex x Genotype** |
| **Right Striatum** | 1.056 ± 0.023 | 0.966 ± 0.057 | 1.017 ± 0.023 | 0.925 ± 0.009 | 8.8 | **0.005** | -4.14 | 0.22 | 0.971 |
| **Left Striatum** | 1.034 ± 0.036 | 0.988 ± 0.052 | 0.978 ± 0.035 | 0.954 ± 0.013 | 3.48 | 0.333 | -4.64 | 0.215 | 0.759 |
| **Cortex** | 1.169 ± 0.023 | 1.182 ± 0.021 | 1.19 ± 0.018 | 1.141 ± 0.024 | 1.54 | 0.39 | -0.85 | 0.638 | 0.135 |
| **Right Hippocampus** | 0.884 ± 0.028 | 0.845 ± 0.041 | 0.781 ± 0.051 | 0.759 ± 0.005 | 3.68 | 0.386 | -12.31 | **0.007** | 0.811 |
| **Left Hippocampus** | 0.939 ± 0.042 | 0.843 ± 0.046 | 0.776 ± 0.047 | 0.82 ± 0.007 | 3.04 | 0.504 | -11.59 | **0.017** | 0.072 |
| **Thalamus** | 0.891 ± 0.029 | 0.822 ± 0.046 | 0.752 ± 0.042 | 0.778 ± 0.012 | 2.64 | 0.526 | -12.03 | **0.007** | 0.164 |
| **Cerebellum** | 0.973 ± 0.084 | 1.087 ± 0.135 | 1.076 ± 0.056 | 1.236 ± 0.04 | -13.4 | 0.111 | 10.93 | 0.142 | 0.79 |
| **Basal Forebrain** | 0.765 ± 0.018 | 0.75 ± 0.031 | 0.989 ± 0.066 | 0.905 ± 0.064 | 5.69 | 0.31 | 20 | **0.00012** | 0.487 |
| **Hypothalamus** | 0.538 ± 0.015 | 0.552 ± 0.006 | 0.531 ± 0.033 | 0.493 ± 0.005 | 2.23 | 0.517 | -6.35 | 0.077 | 0.158 |
| **Right Amygdala** | 0.536 ± 0.008 | 0.609 ± 0.05 | 0.826 ± 0.133 | 0.8 ± 0.034 | -3.45 | 0.747 | 29.68 | **0.001** | 0.496 |
| **Left Amygdala** | 0.478 ± 0.022 | 0.558 ± 0.033 | 0.583 ± 0.063 | 0.605 ± 0.037 | -9.6 | 0.215 | 12.91 | 0.062 | 0.479 |
| **Brainstem** | 0.754 ± 0.011 | 0.739 ± 0.047 | 0.776 ± 0.035 | 0.769 ± 0.038 | 1.42 | 0.758 | 3.4 | 0.455 | 0.906 |
| **Central Gray** | 0.969 ± 0.028 | 0.833 ± 0.066 | 0.822 ± 0.055 | 0.874 ± 0.038 | 4.75 | 0.384 | -6.31 | 0.273 | 0.055 |
| **Superior Colliculus** | 0.887 ± 0.031 | 0.845 ± 0.06 | 0.796 ± 0.051 | 0.838 ± 0.012 | 0.03 | 0.996 | -6.04 | 0.245 | 0.317 |
| **Olfactory Bulb** | 1.413 ± 0.032 | 1.411 ± 0.049 | 1.333 ± 0.02 | 1.309 ± 0.012 | 0.93 | 0.683 | -6.9 | **0.004** | 0.724 |
| **Right Midbrain** | 0.876 ± 0.03 | 0.787 ± 0.054 | 0.706 ± 0.047 | 0.663 ± 0.053 | 8.32 | 0.157 | -21.53 | **0.002** | 0.622 |
| **Left Midbrain** | 0.961 ± 0.06 | 0.809 ± 0.044 | 0.752 ± 0.066 | 0.794 ± 0.02 | 6.4 | 0.278 | -14.44 | **0.027** | 0.054 |
| **Left Inferior Colliculus** | 1.25 ± 0.037 | 1.081 ± 0.097 | 1.067 ± 0.094 | 1.104 ± 0.084 | 5.67 | 0.42 | -7.43 | 0.322 | 0.205 |
| **Right Inferior Colliculus** | 1.257 ± 0.057 | 1.19 ± 0.065 | 0.957 ± 0.08 | 1.032 ± 0.026 | -0.38 | 0.945 | -23.04 | **0.00012** | 0.234 |

Data are expressed as mean ± SEM.
